# Vasorreatividade Coronariana após Reabsorção Total do Absorb BVS no Seguimento de 5 Anos

**DOI:** 10.36660/abc.20190783

**Published:** 2021-02-02

**Authors:** Luis Renier Goncalves-Ramírez, Hipólito Gutíerrez, Fabián Julca, Maximiliano Germán Amado Escañuela, Gretel Varvaro, Ignacio Amat-Santos

**Affiliations:** 1 Hospital de León León Espanha Hospital de León – Cardiologia, León - Espanha; 2 Hospital Clinico Universitario de Valladolid Castilla y León Espanha Hospital Clinico Universitario de Valladolid, Castilla y León - Espanha; 3 Hospital General de Segovia Castilla y León Espanha Hospital General de Segovia, Castilla y León - Espanha; 4 Hospital General de Palencia Río Carrión Castilla y León Espanha Hospital General de Palencia Río Carrión, Castilla y León - Espanha

**Keywords:** Implantes Absorviveis/normas, Intervenção Coronária Percutânea/métodos, Tecidos Suporte/normas, Doença Arterial Coronariana, Infarto do Miocárdio, Recuperação da Função Fisiológica

## Introdução

Os suportes coronarianos bioabsorvíveis foram projetados para prevenir complicações em longo prazo relacionadas ao implante permanente de stents metálicos. O suporte vascular bioabsorvível eluidor de everolimus (Absorb BVS; Abbott Vascular, Santa Clara, Califórnia) foi um dos primeiros suportes vasculares bioabsorvíveis (BVS, do inglês *bioresorbable vascular scaffold*) a ser desenvolvido. O BVS Absorb é uma estrutura feita de ácido poli-L-láctico revestido com polímero poli-DL-láctico, que elui o fármaco antiproliferativo everolimus.^1^ O BVS recebeu a marca CE para o tratamento de doença arterial coronariana em janeiro de 2011 e foi comercializado na maioria dos países europeus em 2012.^2^ Embora bons resultados tenham sido descritos inicialmente,^3^^,^^4^ estudos recentes têm questionado a segurança do dispositivo, sugerindo maior incidência de trombose e infarto do miocárdio.^5^^,^^6^ Além disso, a recuperação estrutural e funcional de segmentos coronários que receberam o suporte após a reabsorção do BVS não foi sistematicamente pesquisada em uma série consecutiva do mundo real.^7^ Descrevemos o caso de um paciente que foi avaliado por angiografia coronariana, tomografia de coerência óptica (TCO) e teste de vasorreatividade coronariana 5 anos após o implante do BVS.

## Relato de caso

**Figura 1:**
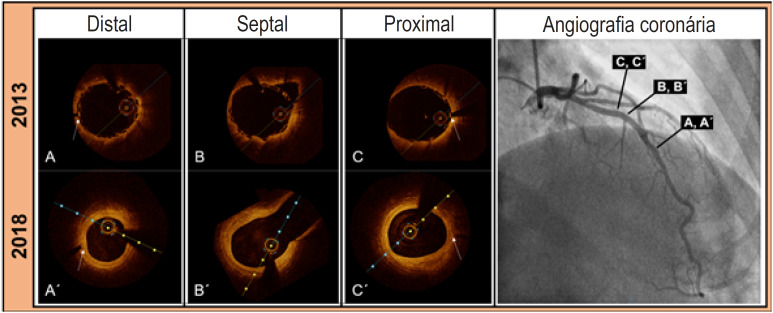
(A, B, C) Pontos no tempo da implantação do Absorb-BVS por TCO. (A´, B´, C´) Achados da TCO no seguimento de 5 anos (mesmo corte transversal). Setas brancas apontam marcadores radiopacos de suportes.

Um homem de 39 anos, ex-fumante, apresentou dor torácica atípica e teste de isquemia inconclusivo. A história pregressa incluía infarto do miocárdio com supradesnivelamento do segmento ST (IAMCSST) há 5 anos, relacionado a doença uniarterial tratada com um dispositivo Absorb BVS de 3,5×28 mm no meio da artéria descendente anterior esquerda (ADA). No tempo presente, o paciente foi submetido a novo cateterismo coronário e não havia evidências de novas lesões ou reestenose. Em seguida, uma tomografia de coerência óptica (TCO) foi realizada sobre o segmento da ADA que recebeu o suporte, mostrando o dispositivo Absorb BVS totalmente reabsorvido, com o desenvolvimento de uma camada neoíntima bem organizada (Figura 1, Vídeo 1).

**Vídeo 1:**
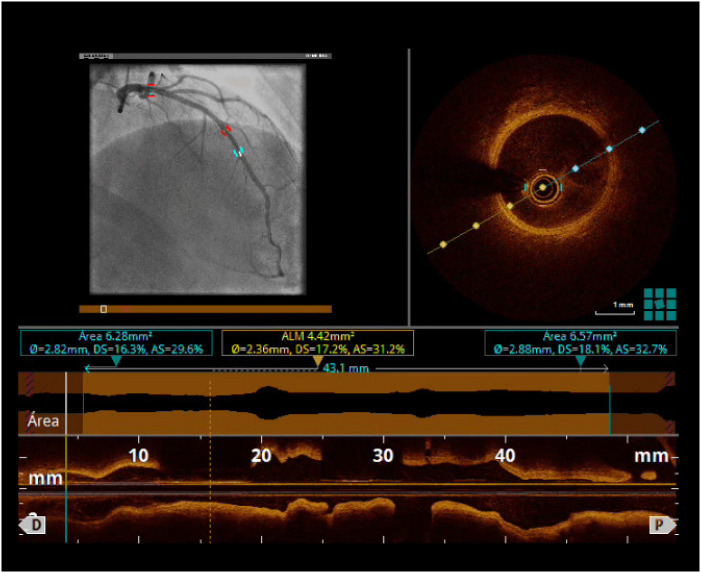
Tomografia de coerência óptica realizada sobre o segmento de andaime de LAD mostrando Absorb BVS totalmente reabsorvido e uma camada neointimal bem organizada. Acesse o vídeo pelo link: http://abccardiol.org/supplementary-material/2021/11601/2019-0783-video1.mp4

**Figura 2:**
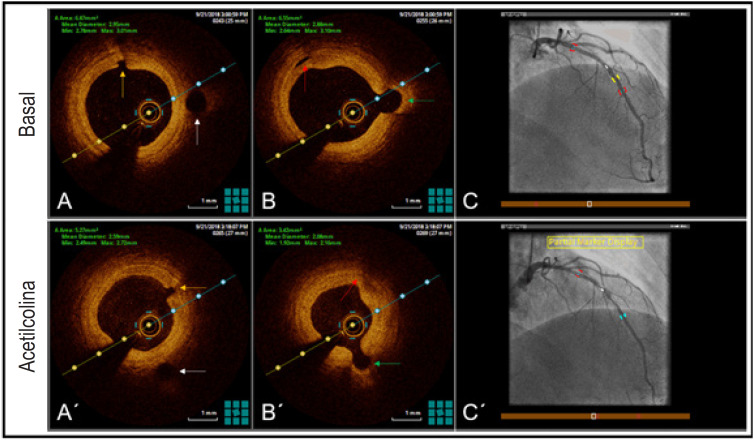
(A, B, C) Imagens basais obtidas por angiografia e TCO. (A´, B´, C´) Achados da angiografia e TCO no mesmo corte transversal após dose máxima de acetilcolina. As setas coloridas indicam os ramos laterais antes e depois do teste.

A vasorreatividade coronária foi avaliada com a administração de acetilcolina intracoronária. Bolus incrementais de acetilcolina foram infundidos (2*µ*g-20*µ*g-100*µ*g) durante 3 minutos cada um, seguido de avaliação eletrocardiográfica, hemodinâmica, angiográfica e TCO da resposta funcional. Na dose máxima de acetilcolina, o paciente desenvolveu dor torácica e espasmo na ADA – incluindo o segmento que recebeu o suporte – conforme observado por ambos, angiografia e TCO (Figura 2, Vídeo 2). Finalmente, um bolo intracoronário (200*µ*g) de nitroglicerina foi administrado para aliviar o espasmo coronário e os sintomas. A repetição da angiografia e da TCO confirmou a resposta vasodilatadora.

**Vídeo 2:**
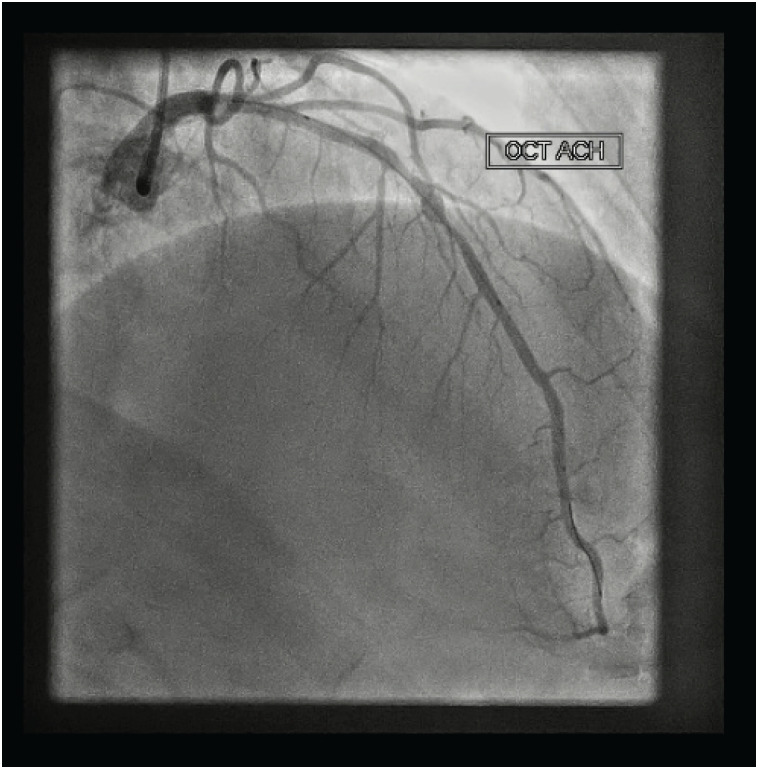
Angiografia coronária mostrando um espasmo LAD -incluindo o segmento de andaime- após o pico da dose de acetilcolina. Acesse o vídeo pelo link: http://abccardiol.org/supplementary-material/2021/11601/2019-0783-video2.mp4

## Discussão

As tecnologias de BVS estão atualmente em destaque no mundo todo devido a uma taxa de eventos adversos de longo prazo maior do que a esperada e ao crescente questionamento quanto à reabsorção completa do dispositivo.^8^ Além disso, dados baseados em evidências de resultados funcionais em longo prazo dos vasos tratados com BVS ainda são escassos.^9^ De fato, se a vasomoção normal *in vivo* é recuperada ou não, permanece sem resposta.

Que seja de nosso conhecimento, este é o primeiro caso que mostra a recuperação morfológica e funcional de segmentos coronários que receberam suporte vascular, 5 anos depois da implantação do dispositivo Absorb BVS em um paciente da vida real. Como já foi descrito anteriormente, o Absorb BVS é finalmente reabsorvido pelo vaso 5 anos após sua implantação, com o desenvolvimento de uma camada rica em sinais visualizada por TCO no segmento que recebeu o suporte, o que corresponde à neoíntima e tecido subjacente,^9^^,^^10^ Por outro lado, a vasoconstrição paradoxal induzida pela acetilcolina e corrigida pela nitroglicerina adiciona informações específicas sobre a recuperação funcional das artérias coronárias que receberam o suporte, sugerindo que o endotélio da neoíntima é sensível a estímulos químicos, mas pode apresentar resposta paradoxal em alguns casos.

## Conclusão

A reabsorção total do Absorb BVS foi encontrada no seguimento de 5 anos. Após a reabsorção do suporte, parece haver um processo adequado de cicatrização do endotélio vascular, com restauração das propriedades morfológicas e funcionais.
