# Supplementary material for: Vasorreatividade Coronariana após Reabsorção Total do Absorb BVS no Seguimento de 5 Anos
Source: Arq Bras Cardiol. 2021 Feb 2;116(2 Suppl 1):8–11. [Article in Portuguese] doi: 10.36660/abc.20190783 (PMC8118635; doi:10.36660/abc.20190783)
Supplement: Translation

# Translation: Coronary Vasoreactivity after Complete Bioresorption of Absorb BVS at 5-Year Follow-Up

**Authors:** Luis Renier Goncalves-Ramírez, Hipólito Gutíerrez, Fabián Julca, Maximiliano Germán Amado Escañuela, Gretel Varvaro, Ignacio Amat-Santos

**Affiliations:** 1 Castilla y León Spain Hospital de León – Cardiologia, León - Spain; 2 Castilla y León Spain Hospital Clinico Universitario de Valladolid, Castilla y León - Spain; 3 Castilla y León Spain Castilla y León Hospital General de Segovia, Castilla y León - Spain; 4 Castilla y León Spain Hospital General de Palencia Río Carrión, Castilla y León - Spain

**Keywords:** Absorbable Implants/standards, Percutaneous Coronary Intervention/methods, Tissue Scaffolds/standards, Coronary Artery Disease, Myocardial Infarction, Recovery of Function

## Introduction

Bioresorbable coronary scaffolds have been designed to prevent long-term complications related to permanent implantation of metallic stents. Everolimus-eluting bioresorbable vascular scaffold (Absorb BVS; Abbott Vascular, Santa Clara, California) was one of the first bioresorbable vascular scaffolds (BVS) to be developed. Absorb BVS is a backbone of Poly-L-lactic acid coated with Poly-DL-lactic polymer, which elutes antiproliferative drug everolimus.^1^ BVS received CE Mark for the treatment of coronary artery disease in January 2011 and it was marketed in most European countries by 2012.^2^ Although good outcomes were initially described,^3^^,^^4^ recent studies have questioned the safety of the device, suggesting a higher incidence of thrombosis and myocardial infarction.^5^^,^^6^ Beyond this, structural and functional recovery of scaffolded coronary segments after BVS resorption has not been systematically searched in a consecutive real-world series.^7^ We describe a case of a patient who was studied by coronary angiography, optical coherence tomography (OCT) and coronary vasoreactivity test 5-year after BVS implantation.

## Case Report

A 39-year-old man, ex-smoker, presented with atypical chest pain and non-conclusive ischemia test. Past history included a ST-segment elevation myocardial infarction (STEMI) 5 years ago, in relation to a single-vessel disease treated with a 3.5×28mm Absorb BVS into mid left anterior descending (LAD). Now, the patient underwent a new coronary catheterization and there was no evidence of new lesions or restenosis. Then, an optical coherence tomography (OCT) was performed over the scaffolded segment of LAD showing fully reabsorbed Absorb BVS with development of a well-organized neointimal layer (Figure 1, Video 1).

**Figure 1:** (A, B, C) Absorb-BVS implantation time-point by OCT. (A´, B´, C´) OCT findings at 5-year follow-up (same cross-section). White arrows point radiopaque markers of scaffolds.

**Video 1:** Optical coherence tomography performed over the scaffolded segment of LAD showing fully reabsorbed Absorb BVS and a well-organized neointimal layer. Access the video at the link: http://abccardiol.org/supplementary-material/2021/11601/2019-0783-video1.mp4

Coronary vasoreactivity was assessed with administration of intracoronary acetylcholine. Incremental bolus of acetylcholine were infused (2*µ*g-20*µ*g-100*µ*g) for 3 minutes each followed by electrocardiographic, hemodynamic, angiographic and OCT evaluation of the functional response. At peak dose of acetylcholine the patient developed chest pain and LAD spasm -including the scaffolded segment- as observed by both, angiography and OCT Figure 2, Video 2. Finally, an intracoronary bolus (200*µ*g) of nitroglycerin was administered in order to relieve coronary spasm and symptoms. Repeated angiography and OCT confirmed the vasodilator response.

**Figure 2:** (A, B, C) Baseline images obtained by angiography and OCT. (A´, B´, C´) Angiography and OCT findings at the same cross-section after maximum dose of acetylcholine. Color arrows point side-branches before and after testing.

**Video 2:** Coronary angiography showing a LAD spasm -including the scaffolded segment- after peak dose of acetylcholine. Access the video at the link: http://abccardiol.org/supplementary-material/2021/11601/2019-0783-video2.mp4

## Discussion

BVS technologies are currently in the spotlight worldwide due to a higher than expected rate of long-term adverse events and growing questions regarding the full resorption of the device.^8^ Moreover, evidence-based data of long-term functional outcomes of the vessels treated with BVS are still scarce.^9^ Indeed, whether *in vivo* normal vasomotion is recovered or not remains unanswered.

To the best of our knowledge, this is the first case that shows both morphological and functional recovery of scaffolded coronary segments after 5-year of Absorb BVS implantation in a real-life patent. As it has been previously described, Absorb BVS is finally reabsorbed by the vessel 5-year after implantation, with a development of a signal-rich layer seen by OCT into the scaffolded segment, which corresponds to neointima and underlying tissue.^9^^,^^10^ On the other hand, paradoxical vasoconstriction induced by acetylcholine and corrected by nitroglycerin adds unique information regarding functional recovery of scaffolded coronary arteries, suggesting that the endothelial from the neointima is sensitive to chemical stimuli but might present paradoxical response in certain cases.

## Conclusion

Fully resorption of Absorb BVS was found at 5-year follow-up. After scaffold resorption, there seems to be an adequate healing process of the vascular endothelium with restoration of the morphological and functional properties.
